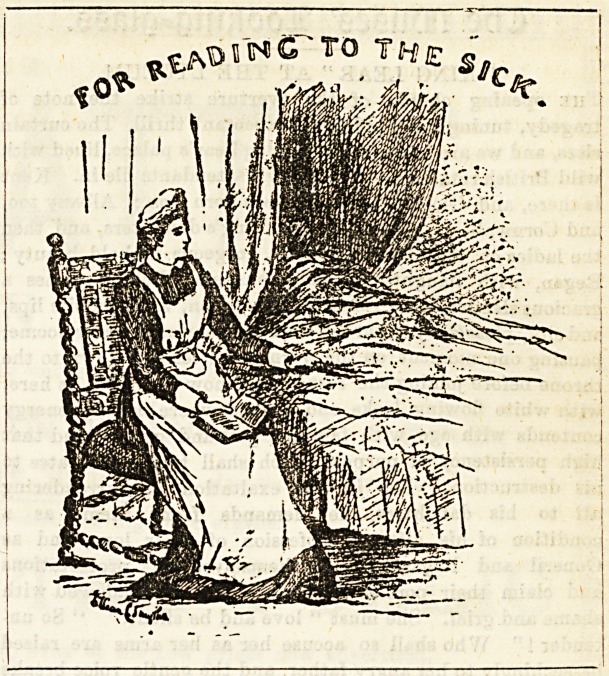# The Hospital Nursing Supplement

**Published:** 1892-11-19

**Authors:** 


"The Hospital" Nursing Mirror.

BEING THE EXTRA NURSING SUPPLEMENT OF "THE HOSPITAL" NEWSPAPER.

[Contributions for this Supplement should be addressed to the Editor, THE HOSPITAL, 140, Strand, London, W.C., and should have the word "Nursing" plainly written in left-hand top corner of the envelope.]

### En Passant.

WHITECHAPEL INFIRMARY.—It is with much pleasure that we learn that the Whitechapel Board of Guardians have resolved to increase the salaries of their infirmary nurses very considerably, so that in this respect the Whitechapel Infirmary will now be placed on a level with the best institutions in the country.

WEIGHED IN THE SCALE.—We should like to warn probationers and others against being over sanguine as to the certainty of obtaining posts for which they apply. The guileless probationer who applies for entrance to a hospital and is accepted on a month's trial is too often bitterly disappointed when she receives intimation that her services as a permanent nurse will not be required. Then again, we have heard several complaints from nurses who, because they have sent up their names and have intimated that they wish to join the Nurses' Co-operation, receive a positive shock when they are politely informed that they are not elected to the roll of nurses. They should bear in mind that there may be others in the field who have stronger claims than they themselves, and that though to be passed over creates a disappointment it is not an injustice.

MISS MARSDEN'S BOOK.—Messrs. Colyer and Colyer, of New Inn Chambers, write: "We are instructed by our client, Miss Kate Marsden, to request you to contradict in your next issue the statement made in your journal of November 12th that Messrs. Sampson Low, Marston, and Co., have withdrawn from the publication of Miss Marsden's book, On Sledge and Horseback to Outcast Siberian Lepers,' and to state the facts—*i.e.*, that the book was 'withdrawn' from Messrs. Sampson Low, Marston, and Co., at the request of the Kate Marsden Leper Fund Committee."

*** Our statement was taken from the *Publishers' Circular*, of 5th inst., on page 536. In reply to our inquiry, Messrs. Sampson Low, Marston, and Co. (Limited) write on 15th inst.: "We beg to say that disputes having arisen as to the interpretation of our terms for publishing the book, it was arranged that we should withdraw from the publication, which we accordingly did, and of course it was absolutely necessary that we should announce the fact.'"

A DOCTOR'S VIEWS ON NURSING.—Dr. Garden read an interesting paper at the Aberdeen Medico-Chirurgical Society, Aberdeen, last week, on Medical Charities, and gave it as his opinion that what had contributed more to start reform in the Aberdeen Medical Charities than anything else was the establishment of the Children's Hospital, and its excellent system of nursing, which first showed what nursing should be. He quoted the Infirmary nursing as being the best system, that is to say, he approves of highly-educated women as sisters in the wards, and nurses of the class to which the nurses there belong; the three years' training now compulsory there Dr. Garden thinks as good as any training in the kingdom. We believe that in the new building accommodation will be provided for a considerable number of nurses over and above what will be required for staffing the hospital, and these will go out private nursing. Dr. Garden seems to share the view with our Commissioner and others that the district nursing should form an integral part of the dispensary work, and as we hear that the Queen's Nurses are now practically working for the dispensary, there seems every probability that a plan of action will be developed to suit everybody. The ideal dispensary in a large town should no doubt have its own staff of resident nurses.

IPSWICH NURSES' HOME.—We are glad to hear that the work of this Home goes forward, and that at a meeting of the subscribers the other day the committee recorded their grateful thanks to the matron and nurses for their unwearied work. The Ipswich home is one of those which is self-supporting in so far as the private nursing of rich folk is concerned, and every penny of the subscriptions and donations goes to the maintenance and development of district nursing. During the influenza epidemic last year, when the nurses were taxed to their uttermost, knowing that in many cases the medicine which their poor patients most needed was good sustaining food, Ipswich rose to the occasion, and £100 were instantly subscribed for extra food and extra nursing.

A MATRON'S WEDDING.—Miss Mary Jane Thompson, who has been matron at Oldham Infirmary for eleven years, was married at All Saints', Manchester, last week, to Mr. John William Penny; her sister, Miss Fanny Thompson, was the only bridesmaid, and Mr. Brown, Mayor of Chester, gave away the bride. Amongst the many presents was a cheque for £50 from the Chairman and Committee of Management of the Infirmary, which was enclosed in a letter of hearty thanks to Miss Thompson for all the good work she had done; the medical staff also sent a cheque, and a letter of congratulation and thanks. Twelve old nurses gave a gold bracelet, while the present nursing staff sent a beautiful Chippendale table.

GLASGOW ROYAL INFIRMARY.—We hear that the new nursing regulations come into force on the tenth of next January. The syllabus of both courses of lectures is very good, and we hope to hear that the lectures and studies on food classification and the essential constituents of diet, will gradually increase in number and importance; practical lectures on ward work and cookery are, we hear, to be given by Mrs. Strong, the Matron. It seems a great pity that any charge should be made for the courses of lectures, for the first course of ten lectures in anatomy, physiology, and hygiene respectively, the fee will be £2 2s.; for the second course of twenty lectures on surgical and medical cases, and the practical lectures, the fee will be £3 3s.

SHORT ITEMS.—We acknowledge with many thanks contributions for next year's fund for the sick bed—8s. 6d. from the Acland Home Oxford Nurses, and 5s. from "A Friend." Also we have received from Nurse Whitsey three pairs of good knitted socks and a knitted vest, for all of which we are very grateful.—Nursing Sister M. Carlisle has been permitted to resign her appointment in the Indian Nursing Service, with effect from March next, on account of ill-health.—The sale of work in aid of the Trained Nurses' Club, Buckingham Street, is fixed for November 26th, and is to be held, by kind permission of Mrs. F. R. Humphreys, at 27, Fellows Road, South Hampstead.—Nurse Isabelia Vine has resigned her post as charge nurse at Lambeth Infirmary, and is taking up private work.—Princess Beatrice and over twenty members of the Balmoral estate and household have been attending St. John Ambulance Association Nursing Classes. Fleet Surgeon Woods is the lecturer.—Mrs. Hodgson Wright, who has supported three district nurses in Halifax free of charge, is about to engage another, who is to pay visits at the rate of sixpence a visit.—There is to be a grand bazaar at Lincoln on November 23rd, and the three following days, in aid of the District Nurses' Fund of the Lincoln Institute for Nurses.

### Lectures for Asylum Attendants.

By William Harding, M.B.

MANAGEMENT OF LUNATICS—(*Continued*).

No insane person was ever yet cured of a delusion by means of reasoning or argument. It is much more probable that an endeavour to do so would have the effect of concentrating the patient's attention upon her false beliefs, and causing the delusions to become more fixed than they were before the attempt. On the whole, the best way is to avoid the subject as much as possible, at the same time giving the patient to understand that the ideas are delusions in your opinion; that they form part of her illness, and that she is only increasing her troubles by dwelling upon them. Sometimes a delusion may arise from the brain exaggerating or misinterpreting messages received from a distant part of the body which is diseased. If this local affection be cured we may hope to see the delusion disappear. In every case, however, the nervous organisation must be weak, and liable to be upset by influences which would have no effect if brought to bear upon a healthy brain. Many patients are fond of bringing forward their troubles and tales of persecution on every occasion. Probably their doing so in some cases provides an outlet for feelings, which, if bottled up, might burst forth in some more unpleasant fashion.

When patients are strong enough every endeavour should be made to induce them to employ themselves. Work is one of our chief curative agents. Of course, if possible, the patient should be got to do useful work, but it should be borne in mind that the object to be aimed at is the good of the patient and not the amount of useful work turned out. The lunatic had better be employed in doing sewing which has all to be unpicked afterwards than be living in idleness. If not occupied at all, it is not to be wondered at if bad habits and violent, or destructive propensities develop themselves. In cases of acute restless excitement in robust individuals, who are too insane to settle to any occupation, it is sometimes necessary to send them for long walks with two nurses, or even to have relays of nurses. This is very exhausting work when the patient is powerful and very lively. These are the cases, especially in acute delirious mania, in which the wet pack is so useful. The method of packing a patient properly can only be taught practically in the wards, but every nurse should be familiar with the process. The excitement which is found in weakly, debilitated individuals requires different treatment. Here nourishing diet and stimulants are essential, and the all-important question of feeding comes in. Food and rest are required, and the patient's recovery to a great extent depends upon careful nursing.

Destructive patients are a trial to a nurse who is careful of her stock. They may be divided roughly into two classes: those who tear up with the wilful intent to destroy, and those who tear and pick at their clothes without being fully conscious of what they are doing. Amongst the latter we may include those cases of restless mania who must be doing something and cannot keep still for a few minutes together. Here we need some means of expending this energy, and in a useful channel if possible. Scrubbing, washing, or any rough household work may be tried, along with open-air exercise. But for all cases of destructiveness continuous supervision and constant attention are necessary. It is not enough that the nurse be present in the ward. She must have eyes and ears for what is going on around her. Because a patient is not quarrelsome and excited she must not conclude that she can leave her unnoticed. It is this habit of leaving such patients alone to their own sweet wills that leads to the formation of dirty and destructive habits. A woman with restless fingers, who is left alone and unnoticed, s very likely to begin picking her dress to pieces. A nurse with a head on her shoulders would observe this tendency and try to find some means of employing the uneasy hands to better purpose, even if it were only to teaze out something of little value. Strong dresses and leather bound clothes may be required in some extreme cases, but constant attention by an intelligent nurse will do much to bring down destruction to a minimum. At night with bad cases the tearing up is nearly unavoidable. The patient who generally requires a single room during her restless hours has nothing to do but tear her bedding or do worse.

It will be necessary occasionally to seclude a patient, that is, to put her into a room alone. In many cases of excitement this allows the patient to calm down without being distracted and upset by those about her. It is the method that one would naturally prefer oneself if agitated and disturbed. When the lunatic is in such a condition that it is thought necessary to keep her apart from the others against her will, and the door is, therefore, locked upon her, this is called seclusion. No patient should ever be secluded without distinct orders to that effect, and on every occasion, no matter for how short a period, the fact of such an occurrence having taken place and its duration should be entered in the ward journal. Seclusion is much less frequently used than it was at one time, and the same remark applies to restraint. Restraint is the term applied to any mechanical appliance which hinders the freedom of the movements of the body. The chief use of restraint nowadays is in cases where it is necessary for surgical reasons, and also sometimes in acutely suicidal individuals. It is now very seldom required, and a nurse may be in an asylum for a long time without seeing it employed. She should, however, know how to put the jacket on if necessary.

Acutely suicidal cases are a great worry to all who have to deal with them. They are a constant strain upon the mind, and the anxiety about them is at times very harassing. The knowledge that you are responsible for the safe keeping of a woman who requires during the whole twenty-four hours to be kept under continuous notice because she is just waiting for an opportunity of injuring herself is very wearing. If, in addition, the patient is, as is frequently the case, uneasy, worrying, and always wishing not to do those things which ought to be done, the life of the nurse in whose care she is placed is not a happy one. In such cases the nurses receive a printed or written notice with the patient, which informs them of the suicidal tendency, and warns them that she must never be permitted to escape from observation.

It will be necessary to examine such patients' clothes each evening at bed time, to see that nothing has been concealed in them which might be used for the purpose of self-injury. There are attempts made by lunatics to smuggle tapes, string, pieces of corset steel, &c., into bed with them, in the hope of being able to effect their purpose under cover of the bed clothes. The teeth of a small buckle have been used for this purpose. Male patients, who have not been thought suicidal, have smuggled sharp instruments into bed and destroyed themselves during the night. Of course, in cases where no warning has been given by the patient in any way such accidents are liable to happen. Patients will frequently etar their clothing into strips, and either attempt strangulation or choking by pushing the pieces into the back of the throat. Handfuls of hair, pulled from the patient's own head, have been used in this way. The fact that injury may be attempted by precipitation down a flight of steps should be borne in mind. I have known a patient to push his head through a window and attempt to cut his throat by sawing his neck against the jagged fragments of glass that remained fixed in the wood. A special word of caution with regard to scissors is needed in the female wards. They are such everyday articles that the chance of their becoming dangerous weapons is apt to be forgotten. The ways in which very suicidal cases will try to effect their purpose are numerous. A woman who appears to have no idea of anything in the world will all the while be pondering methods of putting an end to her existence. The only way of meeting such cases is by continuous and careful supervision. The nurse must not allow her vigilance to be relaxed on any account until the caution given with the patient has been withdrawn. It is not always easy to say whether the suicidal tendency has gone or not, but the nurse must clearly understand how great her responsibility is until the doctor has taken the weight upon his own shoulders by deciding that the patient is sufficiently recovered to have a little more freedom. In coming to such a decision the opinion of an experienced nurse is always of great value.

(*To be continued.*)

### Nurses' Bookshelf.

### HOUSEHOLD NURSING.*

A useful handbook of comfortable dimensions reaches us entitled "Household Nursing," by Dr. J. O. Tunstall, late senior resident medical officer at the Birmingham Infirmary. It is intended for a guide to the amateur nurse who, when circumstances prevent the employment of a trained nurse, is obliged to tend a sick friend or relative. As the author says in his introduction, it is quite impossible under many conditions for the untrained nurse, however devoted and willing, to take the place of her professional sister; but it is better that what she can do she should do rightly, and the book is certainly full of useful suggestions for her. The general observations on the ventilation and general management of a sick room are very clearly given, and the remarks on "bed-making," a point on which the amateur nurse is generally painfully ignorant, will, we hope, be the means of saving many restless nights to weary patients; every woman ought to learn how to change the lower bed-clothes without disturbing a patient and how to use the inestimable draw-sheet. Chapter II. contains directions for the special nursing of infectious and other cases, the theory of disinfection, and a review of disinfectants; while Chapter III. gives much useful information concerning food in disease and food for infants, while the appendix at the end of the book contains clear and simple recipes for the preparation of invalid diet. The author has avoided the error of being too technical or of pre-supposing knowledge on the part of his readers.

* "Household Nursing." By John Ogle Tunstall, M.D. (London; T. Fisher Unwin.)

### Appointments.

Brighton College.—Miss Wilkes, who has been charge nurse at the hospital, Fir Vale, Sheffield, has been appointed Matron to Brighton College.

St. Saviour's Infirmary, East Dulwich.—The two new sisters who have recently been appointed here are Miss J. Hickman, who was trained at the General Infirmary, Nottingham, and who was afterwards charge nurse at the Carmarthen Infirmary, and then Matron at the Government Hospital, King William's Town, S. Africa; and Miss M. W. McDonall, who was trained at the London Hospital, and has since been head nurse at Tynemouth Infirmary.

Indian Nursing Service.—Miss Mary Bartlett has been appointed nursing sister on this staff, and sails in the "Serapis" on the 24th inst. Miss Bartlett trained for a year at Brighton Children's Hospital, she then went to Barnwood House, Gloucester, for six months, after which she went to Westminster Hospital, and gained her three years' certificate. Miss Mary Bartlett also gained the Clapham Maternity Certificate, and holds a certificate as an efficient masseuse from Dr. Stretch Dowse. Miss Bartlett has been working for the Nurses' Co-operation, and holds excellent testimonials.

### The R.B.N.A. and the Lords' Committee's Report.

Dr. W. Bezley Thorne, Hon. Secretary Royal British Nurses' Association, writes: From the time when the petition of the Royal British Nurses' Association for a charter of incorporation was laid before the Privy Council. it has been the desire of the members of the Association, in accordance with a well-recognised and honoured custom, to avoid such public utterances as might invade either the province of that tribunal or the sphere of the learned advocates to whom the duties of defence and opposition have been committed. As, however, an article in your last issue, by challenging the accuracy of a statement of mine, and suggesting the propriety of an apology, converts, to a certain extent, a public question into a personal one, I take the liberty of departing, for once, from the salutary custom to which I have alluded. The statement which you challenge is correctly quoted in your article, and, far from withdrawing or modifying it, I repeat with emphasis that the Committee of the House of Lords on Hospitals did not in their third or any other report pronounce adversely on the system of registration initiated by the Association of which I have the honour to be one of the executive officers. My assertion is proved by the "conclusions" which alone express the opinions of the Committee. That body, beyond all question, deliberately abstained from expressing any opinion on the subject whatever, for the reason that neither party had been officially cited before them or exhaustively heard, as any reader of the report may readily ascertain. Your contention to the contrary, and assertion that the Committee decided that "the Royal British Nurses' Association register . . . . will be no protection to the public, though it would tend to reduce all nurses to one common level," is definitely contrary to the fact, for no such passage or any other bearing on the subject is to be found in the "conclusions." It seems to me, therefore, that if any apology be due, it is from those who, doubtless through inadvertence, have on two separate occasions given to their readers, as the pronouncement of a tribunal, the pleadings of their own advocates. Probably it is to be attributed to the same defect of mental concentration that you make your quotation from my speech to end at the point at which I proceeded to say that my statement which you call in question was based on the authority of the President of the Lords' Committee himself. And now I will only add with reference to the voting of the Committee on the question whether they should express an opinion on the value of the register of the British Nurses' Association and support the petition of the Association for incorporation, that however grateful the Association must be to the Lords who were prepared to plead its cause, I am in a position to state positively that the Committee voted as they did solely on the ground which I have already indicated, and that if they had come to a decision adverse to the aims of the Association they would unhesitatingly have embodied an expression to that effect in their carefully formulated "conclusions."

*** Dr. Thorne's ingenious contention that the Lords' Report and the conclusions contained in it are not one and the same document, cannot be admitted for a moment. In fact, the conclusions occupy a relatively small portion of the Report, of which they form but a part, though, no doubt, an important part. Does Dr. Thorne seriously contend that the effect of paragraphs 508 to 513 of the Lords' Report is not to show that the registration of nurses as proposed by the Royal British Nurses' Association "will be no protection to the public (paragraph 512), though it would tend to reduce all nurses to one common level" (paragraphs 510 and 511)? Dr. Thorne's further contention that the Lords voted as they did solely on the ground that they were not prepared to express an opinion on the value of the R.B.N.A. register, or they would have embodied an expression to that effect in their conclusions is negatived by a reference to "the draft report by the chairman" and by comparing it with "the report" as finally adopted. In the "draft report" the conclusions included a paragraph (598), which we printed last week, in favour of the R.B.N.A. proposals. This conclusion, after due consideration and postponement, was ultimately rejected on a division by six votes to two. If a formal vote taken by a committee on a point like this is not a deliberate expression of its opinion on the subject, will Dr. Thorne kindly tell us what is? This vote is, no doubt, an unpalatable fact for Dr. Thorne's Association, but would it not be franker and better to admit its consequences whilst endeavouring to save the cause from the effects of so damaging a blow? No amount of verbal persiflage can alter the fact that the Lords did reject the registration proposals of the R.B.N.A., and we still hope an apology will be forthcoming from Dr. Thorne, whose good faith and zeal on behalf of his Association we most cordially recognise.—Ed. T. H.

### Notes from Australia.

(*By Our Own Correspondent.*)

Melbourne, October 4th, 1892.

Money, or the want of it, is the root of all evil, and we, over here, are feeling keenly alive to the fact that financial troubles affect the poor hospitals to a very serious extent. The case of the Children's Hospital is particularly hard, and how to keep pace with the constant demand for relief is a difficult problem. When the boom was at its height the institution in Pelham Street benefited largely by a generous public's subscription, and the balance-sheet of 1889 to 1890 showed a credit balance opening of £1,900, and, after all expenses had been paid, closed with one of £2,500. This state of affairs had, however, anything but a desirable result, for the Government subsidy, which has been £1,000 a year, was cut down to a tenth of that sum. The public subscriptions followed suit, and now, at the end of the next financial year, if no help is forthcoming, there will be a debt of nearly a thousand pounds. If the Melbourne people really want the hospital to do good they must not only maintain, but increase it; as it is, the nurses' work on cramped up for want of better accommodation, and the seventy-six beds are never empty.

Two meetings have been held with a view to forming an Australian Nurses' Association, "to unite all qualified nurses in membership of a recognised profession," according to the circular, but we old-fashioned people, who know how recent are any really good nursing schemes out here, and how very few really first-class nurses are as yet in existence, think the whole thing premature and unnecessary, and wonder why people nowadays rush into everything in such a hurry; insurance and pension funds are also to be formed. Personally speaking, I am content with my certificate and testimonials from my Alma Mater, and any five shillings I have to spare are going to be saved with much care. It seems to me that we are only on the very doorstep of efficient nursing—there is a great deal of smattering, and not enough real study.

We are having a great cremation movement, and many meetings and people are saying that their feelings are being very much hurt; but we are decidedly behind the times here on this matter, and many of us wish that the day may not be far distant when we shall arrive at some definite conclusion anent the advisability of cremating the bodies of all who have died from infectious diseases. People will be so personal on these matters, and will not content themselves with threshing them out on the ground of public health alone.

Dr. Tyson, of United States renown, has opened a "Drink-cure Sanatorium" at St. Kilda; it is a most lovely house and very luxurious. Somebody told us the other day that two of the once habitual drunkards are now in charge of the wine and spirit department! But why there is any intoxicating liquor to be taken care of seems a pertinent question.

The Inter-Colonial Medical Congress has been held at Sydney; quite the most interesting papers were those on the plea of insanity in criminal trials, especially those of Dr. Springthorpe and Dr. Manning. It was finally moved: "That in view of the fact that the present recognized legal test for insanity is false in theory and unsatisfactory in practice, and that legal authorities have expressed a desire to obtain some authoritative expression of medical opinion upon the subject, this section submits for ratification by the general congress the following resolutions: 1. That it is impossible to frame any single test for insanity in criminal cases, which is capable of general application. 2. That for criminal cases it would be in accord with the present medical opinion to define insanity as a disease of the brain affecting the intellect, the emotions, and the will, and not immediately induced by default of the individual, leaving the two following questions for the jury: (*a*) Has the accused such disease? (*b*) Was his act the outcome of such disease?" Which, after all, is very much the same difficulty as that in which a jury is placed now.

### Everybody's Opinion.

[*Correspondence on all subjects is invited, but we cannot in any way be responsible for the opinions expressed by our correspondents. No communications can be entertained if the name and address of the correspondent is not given, or unless one side of the paper only be written on.*]

### HEALTH CLASSES FOR THE PEOPLE.

"A Correspondent" sends us the following scheme for the instruction of lecturers on health, &c., in rural districts: If the technical education scheme is to prove successful in rural districts, teachers of the laws of health, &c., must be found, who are both themselves thoroughly conversant with what they are going to teach, and able to impart knowledge to country folks, so that they may understand and may feel that the teaching is what they can and ought to put into practice in their own homes. The mistake that is too often made is that these people are not taught to make the best of what they have at hand, and to improve to the utmost the conditions under which they are compelled to live. Instead of this, expensive appliances and impracticable methods are recommended which are far beyond their means, and hence the country folk only too frequently say, "It is all very well for the gentry, but it is not for the likes o' me." In consequence, there is little or no result from much of the teaching, and no improvement whatever is seen in rural cottages. It is with a view to remedy these defects in teaching that a scheme has been originated by a number of ladies experienced in the requirements of country districts to give a thorough theoretical and practical training. For this purpose classes have been started, and it is intended to keep especially in view the requirements of these teachers, and above all to make the instruction practical, and at the same time thorough. The course will include ten lectures on each of the following: (*a*) Sanitation, (*b*) Elementary Anatomy and Physiology, (*c*) Personal and Domestic Hygiene. Whilst courses (*a*) and (*b*) are to supply theoretical knowledge, course (*c*) will be of a popular nature, to show how the laws of health can be taught in a simple but scientific way, easily understood by the comparatively uneducated. In addition, frequent examinations will be held, and each student will be expected to prepare and write lectures to be read and criticised, and special importance will be attached to appropriate illustrations, and to simple, practical experiments to make the lectures as interesting as possible. The full preparation will occupy three months, but a student can enter for any single course. It is hoped that these classes will be recognised by a well-known examining body, who will grant a certificate of proficiency in the above subjects to those who satisfy the examiners. Ladies who wish to take up this lecturing, but are not trained nurses, are strongly recommended, if possible, to attend instruction in Ambulance and Nursing, in addition to the above courses, as these are not included in the present scheme. Further information can be obtained by writing to Miss Lamport, 55, Burton Crescent, W.C.

### ASYLUM NURSES.

"Another Asylum Nurse" writes: Several articles and letters have appeared in your paper of late, in which statements were made which were anything but complimentary to asylum nurses. I feel constrained to take the liberty of informing the writers and the editor himself, that in the asylum where I am a nurse, the nurses are a superior class of women, and have never been employed either in hotels or lodging houses, neither do the attendants belong to the class who are too lazy to work otherwise. I can assure you such would not find employment here. Neither the physician, superintendent, nor our loved and esteemed matron would engage anyone whose character could not bear the strictest investigation. Our asylum is conducted on the highest moral principle, and I do not think it either fair or just to those who are trying to raise the standard of asylum nurses, to publish these unfavourable articles, granting they were true when written twenty years ago. If the writer who wrote lately would like to see the sick or dying lunatics properly nursed, he has just to come here and see them. The duties of asylum nurses being so much more arduous and trying than any other nursing, argue more pith, courage, and self-denial rather than less and we trust the day is not far distant when asylum nurses will be looked upon with the respect they merit. One of our number always assures us that the day is coming.

[See report of our Special Commissioner on Berrywood Asylum.—Ed.]

### "WHERE TO LIVE."

"E. B." writes as follows; perhaps others will help her: "Can you kindly tell me through your paper whether there are any other residences or flats on the same principle as the York residences, York Street, Portman Square, but with less rent, where I could get a fair-sized, unfurnished room, and board if I wished when disengaged. I have the necessary furniture, and the older I get the more I enjoy possessing my own home; but I find unfurnished lodgings at a reasonable price are too horrid to think of. I shall be very grateful for any advice which will help me to find what I require. I do not think I ought to spend more than £20 a year in rent for a room—£5 a quarter—because as I am a "private" nurse my income is precarious. I should like to be within easy distance of Vere Street. Do you know anything about those buildings, opened November 1st by the Duke of Westminster, forthe working classes, near Grosvenor Square, W.C.? Surely it would be a charity if someone would build a nice comfortable quiet place where quiet people could provide a home at moderate terms."

### Wants and Workers.

*Convalescent H me.*—Can anybody kindly give me an address where a middle-aged gentleman (in reduced circumstances) can go for a few weeks after a bad illness, Bournemouth, Ventnor, or the south coast?—*M. B.*

*Music Wanted.*—The Matron of the Southend Victoria Hospital, Essex, will gratefully receive an old piano or musical box for the amusement of her patients, whose days of convalescence become tedious and wearisome, and which would be conside ably lessened by the amusement it would afford.

### "FRAGRANCE."

There is one thing we none of us like, and that is, to be forgotten when we are away; though still worse is it to know people are saying, "So and so is such a grumbler it is a relief he or she is gone." There is, however, a way in which our lives may leave a memory full of fragrance, and that is by being kind, and gentle, and obliging to all about us. We all value the rich perfumes of nature, and drink in with rapture the pure scents of the rose, the lily, and the violet, as well as that from the leaves of the orange, thyme, and mint, or from the fruit of the bergamot, the seed of the anice and carraway, and the wood of the cedar, bark, and cinnamon. It is the sun with its warmth and light which brings all these delights to perfection, and it is when our Lord Jesus Christ is allowed by us to take His right place in our hearts, and the Holy Spirit to dress and keep those precious gardens of our souls, that we shall breathe out from flower and fruit, that is from our pleasant actions and our good deeds, the influence of Christ's presence. The presence of God in our hearts when we are really praying to be like Him, will be as free and natural to us as fragrance is to the flowers. But our Heavenly Father sees not as man sees, and one thing which presents itself to His sight is that many seem unable to give out the best that we have in us, and so He presses His hand upon us to make us bring forth the full odour of which we are capable. Who has not heard of the processes in the South, where the violet, the tuberose, the orange flower, &c., are crushed together till their whole scent is exhausted, and then the perfume goes throughout the world as the valuable "eau de Cologne"? Sickness is to us what the press is to the flowers; it finds out what our real selves are, whether we are wanting to be truly good, or only just good enough to escape punishment. Our Saviour will crush the power of sin out of our hearts and calm our troubled breasts, and then we shall send up prayers and praises, like the smoke of the incense which ascended of old from the altar of God, and found its way into the holiest of all. The scene in the house of Bethany, where the woman broke the alabaster box over the feet of our Lord, has passed away, but not the memory of it; its fragrance is as powerful as ever in the Gospels, and by them throughout the whole world. She gave of her best to her best. We may be poor and weak and lonely, and not very clever, but every act of our lives done from God and done for Him, will receive the approbation which was bestowed on the sinner, "She hath done what she could." In the words of a modern writer, "Fragrance is caused by a forgiving spirit coming from a rejoicing heart, which is agreeable in its manner, gentle in its tone, restrained in its actions, attentive to God's call to right, noble in purity and truth, conscientious in its secret life; and this life endears itself to those around, and leaves ever a fragrance of Christ.

### The Muses' Looking-glass.

### "KING LEAR" AT THE LYCEUM.

The opening chords of the overture strike the note of tragedy, tuning the mind to an expectant thrill. The curtain rises, and we are in the hall of King Lear's palace, lined with wild British retainers. The King's attendants file in. Kent is there, and Gloster, with his base-born son; Albany too, and Cornwall, as suitors for the King's daughters, and then the ladies of the Court—Goneril, gorgeous in bold beauty; Regan, with haughty self-consciousness. Behind comes a gracious slender form robed in pale green, with mobile lips, and shy, pleased eyes, turned round in answer to her welcome, pausing one moment on the worn steps leading down to the throne before joining the rest. And now the King is here, with white flowing locks and a face where kingly energy contends with age, with familiar gait and gesture and that high persistency of purpose which shall tempt the fates to his destruction. For in the exaltation of surrendering all to his daughters, he demands from them, as a condition of his gifts, a profession of their love, and as Goneril and Regan spend themselves in protestations and claim their portion, Cordelia's face is shadowed with shame and grief. She must "love and be silent." "So untender!" Who shall so accuse her as her arms are raised beseechingly to her angry father, and the gentle voice breaks at the word "love" in her plea of dutiful affection? But all the past years of loving service are forgotten in the burst of passion which greets her reticence. Banishment, poverty, reiterated shame is heaped on the head bowed with repressed anguish while the King's inarticulate wrath gives the first hint of an over-wrought brain. Then, as she hides her face in the abandonment of grief while the royal train passes out, the Fool stoops with quick, reverent motion, and touches the hem of her gown with his lips. All hearts go with the act of homage, and not many eyes are dry.

And now the King, with retinue of knights and faithful Fool, and the disguised Kent, is in the castle of Goneril. And her cold, measured insolence is opposed to Lear's rising fury. Very terrible is the outbreak of the outraged King, realising with convulsive struggle against tears the powerlessness of his wrath. It culminates in the terrible curse with which he turns from her to seek a securer refuge with Regan.

We follow him to the courtyard of the Duke of Gloster's castle, whither Regan has retreated at the news of his approach. A fair morning is spreading over the wide landscape. Space forbids our lingering over a scene yielding to none in the fine gradations of pathos and passion. Mr. Haviland as the Fool is beyond all praise—in his tenderness and the humour which hides a tear affording the only relief to the growing tension of the situation. To him the King turns when coldly repulsed by Regan, hotly chided by Goneril, he is swept out of himself by the wild current of his passion. The *hysterica passio* has conquered, and struggling with ever fainter utterance against the rising tears, he sinks sobbing out at length on the arm of his follower, "O, Fool, I shall go mad!"

Thick darkness; a lonely heath, with beyond a low range of hills lit up by frequent lightning; a night of horror, where the howling wind, the ceaseless swish of rain, and sudden thunderclap produce wild discord; and in the midst the King, in the extremity of his despair. We see none but him. The Fool may try with loving jest to ease the sick heart; the feigned madman brings his wild antics to add yet another note of horror; Kent and Gloster exhaust their entreaties; but Lear is still alone. That frail form, grandly wrestling with fate, slowly conquered, finally subdued to madness stands out in supreme isolation. It is the wreck of a mind which is here enacted, of a great mind, for Lear is never greater than in the dignity of his utter abandonment. Slowly in the warmth of the farmhouse, whither they lead him, a torpor steals upon him, and at the gentler tone and lingering intonation with which he stretches his limbs by the fire the tension slowly relaxes—the worst is over.

It will be remembered that the King is conveyed to Dover to join Cordelia, who is advancing with a French army to his rescue. Escaping there from custody, he encounters Gloster on Dover Cliff, a scene of singular beauty, with its distant view of rock and sea. A change has come over his mood. Before, in the storm, reason was in possession, and battled fiercely with the rising madness. Now she lies almost wholly obscured, yet streaming, as it were, in broken rays through the disordered brain. "Every inch a King," even so. Yes, even in the mad laugh with which he totters from the stage, though attaining the highest point of realism, Mr. Irving saves his beautiful conception of the part from any touch of the trivial or base.

Then, after an interval spent amid the clamour of arms, the mean stratagems of the sisters, and the treachery of Edmund, we approach the tent where the sick King, royally robed once more, lies wrapped in sleep. The light falls across his face, hushed in a white stillness as of death itself. And over him hangs Cordelia, winning him back to life with softest kiss and reverent, tender voice; a queen, whose majesty is all absorbed in the devotion of a most loving daughter. A wonderful awakening to life. Slowly, very slowly, the light of reason, faint and flickering at first, grows clearer, till the faltering voice ventures at length in answer to that quivering face and those imploring arms, "Do not laugh at me; for as I am a man, I think this lady to be my child Cordelia." No one who has ever heard it will forget the low thrill, with underlying tears, of Cordelia's "And so I am, I am," followed by the shuddering, low drawn sobs as the father rests in the shelter of her protecting arms.

After this the end can be borne. One more glimpse we have of the fond, worshipping father smiling at his bonds as Edmund dismisses him to prison with Cordelia, "We two alone will sing like birds in the cage." And then comes the final moment of anguish when Lear bears in the dead Cordelia, and that great wounded heart finally succumbs. Here is no violence of grief. Only a slow loosening, one by one, of the cords of life in presence of that motionless grey form—the soul she had recalled to its seat of reason fading away after her in faintly-drawn whispers till all is silence.

### Notes and Queries.

### Queries.

(26) *Lecture on Cholera.*—Can I get the lecture on cholera read before the Health Society?—*M. M. W.*

(27) *List of Books on Massage.*—Will you give me the names of some books on massage?—*L. A. P.*

(28) *Where to send Dolls.*—Where shall I send two dozen dolls and a box of Christmas cards?—*E. N. M.*

(29) *Lectures on Cholera.*—Will there be any lectures on cholera during the winter?—*A. P. R.*

(30) *A District Nurse's Bag.*—Can anyone tell me the best place to procure a district nurse's bag of light weight and with suitable fittings? —*A. S.*

### Answers.

(26) *Lecture on Cholera* (*M. M. W.*).—The lecture was read before the National Health Society, Berners Street. You had better apply there and see if it is printed.

(27) *List of Books on Massage* (*L. A. P.*).—"Primer of the Art of Massage," by Stretch Dowse (Simpkin, Marshall), Murrell's "Masso-Therapeutics" (H. K. Lewis), Ostrom's "Massage, and the Swedish Movement" (H. K. Lewis), "Nurses' Guide to Massage," by Samuel Hyde (John Heywood), Mrs. Creighton Hale's "Art of Massage" (Scientific Press).

(28) *Dolls and Christmas Cards* (*E. N. M.*).—If you will trust us to find a welcome home for the dolls, we shall be very grateful for them for our Christmas parcels.

(29) *Lectures on Cholera* (*A. P. R.*).—We will inquire for you, but write to the Trained Nurses' Club, and see if they mean to have any.

(30) *A District Nurse's Bag* (*A. S.*).—We should advise your going to Messrs. Bailey, 38, Oxford Street, W, Messrs. Hockin Wilson, 186, Tottenham Court Road, and Mr. W. R. Stacy, 4, Newgate Street, they all keep bags of every sort, and at one or other of the firms you are sure to get exactly what you require for your own particular needs.

## Figures and Tables

**Figure f1:**